# Neural mass modeling for the masses: Democratizing access to whole-brain biophysical modeling with FastDMF

**DOI:** 10.1162/netn_a_00410

**Published:** 2024-12-10

**Authors:** Rubén Herzog, Pedro A. M. Mediano, Fernando E. Rosas, Andrea I. Luppi, Yonatan Sanz-Perl, Enzo Tagliazucchi, Morten L. Kringelbach, Rodrigo Cofré, Gustavo Deco

**Affiliations:** Sorbonne Universite, Institut du Cerveau - Paris Brain Institute - ICM, Inserm, CNRS, Paris, France; Department of Computing, Imperial College London, London, UK; Department of Psychology, University of Cambridge, Cambridge, UK; Department of Informatics, University of Sussex, Brighton, UK; Sussex Centre for Consciousness Science and Sussex AI, University of Sussex, Brighton, UK; Centre for Psychedelic Research and Centre for Complexity Science, Department of Brain Science, Imperial College London, London, UK; Centre for Eudaimonia and Human Flourishing, Linacre College, University of Oxford, Oxford, UK; Department of Clinical Neurosciences and Division of Anaesthesia, University of Cambridge, Cambridge, UK; St John’s College, University of Cambridge, Cambridge, UK; Information Engineering Division, University of Cambridge, Cambridge, UK; Buenos Aires Physics Institute and Physics Department, University of Buenos Aires, Buenos Aires, Argentina; Universidad de San Andres, Buenos Aires, Argentina; Institut du Cerveau et de la Moelle epiniere (ICM), Paris, France; Institucio Catalana de la Recerca i Estudis Avancats (ICREA), Barcelona, Spain; Latin American Brain Health Institute (BrainLat), Universidad Adolfo Ibañez, Santiago, Chile; Department of Psychiatry, University of Oxford, Oxford, UK; Center for Music in the Brain, Department of Clinical Medicine, Aarhus University, Aarhus, Denmark; Institute of Neuroscience (NeuroPSI), Paris-Saclay University, Centre National de la Recherche Scientifique (CNRS), Gif-sur-Yvette, France; Center for Brain and Cognition, Computational Neuroscience Group, Department of Information and Communication Technologies, Universitat Pompeu Fabra, Barcelona, Spain

**Keywords:** Whole-brain model, Mean-field model, Computational model, Local inhibition

## Abstract

Different whole-brain computational models have been recently developed to investigate hypotheses related to brain mechanisms. Among these, the Dynamic Mean Field (DMF) model is particularly attractive, combining a biophysically realistic model that is scaled up via a mean-field approach and multimodal imaging data. However, an important barrier to the widespread usage of the DMF model is that current implementations are computationally expensive, supporting only simulations on brain parcellations that consider less than 100 brain regions. Here, we introduce an efficient and accessible implementation of the DMF model: the FastDMF. By leveraging analytical and numerical advances—including a novel estimation of the feedback inhibition control parameter and a Bayesian optimization algorithm—the FastDMF circumvents various computational bottlenecks of previous implementations, improving interpretability, performance, and memory use. Furthermore, these advances allow the FastDMF to increase the number of simulated regions by one order of magnitude, as confirmed by the good fit to fMRI data parcellated at 90 and 1,000 regions. These advances open the way to the widespread use of biophysically grounded whole-brain models for investigating the interplay between anatomy, function, and brain dynamics and to identify mechanistic explanations of recent results obtained from fine-grained neuroimaging recordings.

## INTRODUCTION

Recent advances in noninvasive brain imaging technology provide a fertile ground to investigate how the anatomical structure of the brain shapes complex neural dynamics, in both healthy and pathological conditions. The high versatility of the available imaging modalities has triggered a plethora of research efforts, which in turn have delivered important advances in human neuroscience (Breakspear, 2017; Deco & Kringelbach, 2020; Einevoll et al., 2019; Munn, Müller, Wainstein, & Shine, 2021). To move beyond correlational inference and toward causal understanding, such tools can be combined with causal manipulations of brain activity induced by tasks, pharmacology, or noninvasive brain stimulation, for example, transcranial magnetic stimulation (Hallett, 2007; O’Shea & Walsh, 2007), transcranial direct-current stimulation (Brunoni et al., 2012; Stagg & Nitsche, 2011), direct cortical stimulation in surgical patients (Fox et al., 2020), or the study of other neuropsychiatric patients (Bisiach & Luzzatti, 1978; Damasio et al., 1994; Scoville & Milner, 1957; Weiskrantz, 1990). However, ethical and practical considerations mean that such approaches fall short of the exquisitely fine-grained experimental control that is nowadays available, which has provided fundamental new insights about the causal mechanisms of brain function (Miesenböck, 2011; Siddiqi, Kording, Parvizi, & Fox, 2022).

One attractive approach to bridge the gap between neuroimaging data and causal mechanisms in humans is whole-brain modeling, where neurobiologically inspired models informed by multimodal neuroimaging empirical data are used to reverse-engineer various aspects of brain function. Whole-brain models typically consist of differential equations describing local dynamics of neuronal populations, coupled through a network of empirically derived anatomical connections (e.g., tract-tracing data from animals or diffusion tensor imaging (DTI) data from humans) (Cabral, Kringelbach, & Deco, 2017; Coronel-Oliveros, Cofré, & Orio, 2021; Deco, Cruzat, et al., 2018; Deco et al., 2014; Deco, Tononi, Boly, & Kringelbach, 2015; Shine et al., 2021). After appropriate tuning, such models make it possible to simulate plausible brain dynamics, which can then be compared with empirical brain dynamics (as measured, e.g., with functional magnetic resonance imaging (fMRI) or electroencephalography (EEG)), thereby allowing researchers to investigate the relationship between anatomical connectivity and brain activity and local network dynamics, providing a valuable tool to bridge scales and narrow down the space of mechanistic explanations compatible with empirical findings (Cabral et al., 2017; Cofré et al., 2020; Deco, Cabral, et al., 2018; Deco et al., 2014, 2019; Deco, Cruzat, et al., 2018; Herzog et al., 2023; Ipiña et al., 2020; Kringelbach et al., 2020; Luppi, Cabral, et al., 2022). Whole-brain models also provide an ethical and inexpensive “digital scalpel” that allows researchers to explore the counterfactual consequences of modifying structural or dynamical aspects of the brain, some of which would be hard—if not entirely impossible—to assess experimentally. This ability to assess counterfactuals makes whole-brain modeling a promising tool to deepen our understanding of the mechanisms behind brain disorders and to explore novel therapeutic interventions, including drug treatments or brain stimulation (Deco, Cabral, et al., 2018; Deco et al., 2019; Deco & Kringelbach, 2014; Luppi et al., 2023; Sanz Perl et al., 2021).

The literature offers a wide range of whole-brain models, with some of them being supported on platforms such as *The Virtual Brain* (Ritter, Schirner, McIntosh, & Jirsa, 2013) or *neurolib* (Cakan, Jajcay, & Obermayer, 2023). Available models can be classified according to their trade-off between conceptual simplicity and neurobiological realism. On one side of this spectrum, models prioritize local equations that reproduce the empirical data with a minimal number of assumptions, and fine-tuning of parameters, thus, are useful to reveal general dynamical principles (e.g., simulating brain areas as nonlinear oscillators near the transition to global synchronization) (Breakspear, Heitmann, & Daffertshofer, 2010; Deco et al., 2015, 2019; López-González et al., 2021; Lord, Stevner, Deco, & Kringelbach, 2017). On the other side of the spectrum, models are more complex but they can be interpreted in terms of the biology and biophysics of neurons (e.g., modeling brain areas as balanced excitatory/inhibitory neural populations) (Abeysuriya et al., 2018; Deco, Cruzat, et al., 2018; Deco et al., 2014; Vattikonda, Surampudi, Banerjee, Deco, & Roy, 2016). In general, the former are faster to simulate and easier to tune and control, while the latter provides more realistic explanations and can incorporate additional neurobiological information, such as receptor densities obtained using positron emission tomography (Deco, Cruzat, et al., 2018; Herzog et al., 2023; Mindlin et al., 2023) or transcriptomic data (Burt et al., 2021).

**Figure 1.**
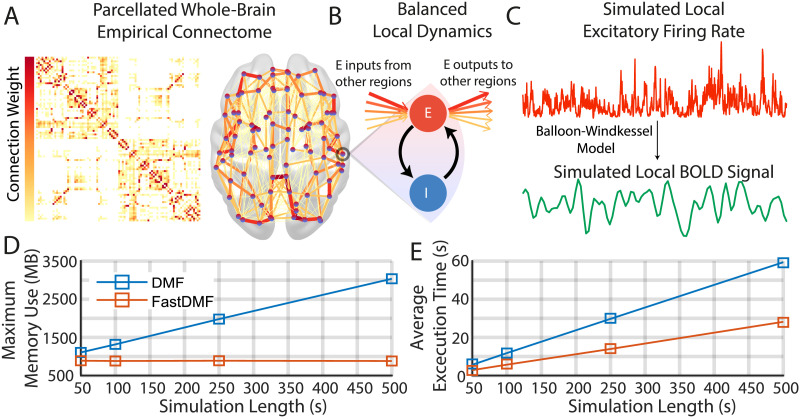
Simulations using the FastDMF model. (A) Brain regions are defined by a parcellation (here, the AAL90), and their connections are empirically obtained from DTI (see the Materials and Methods section for details). In the schematic brain, circles are brain regions, and connections between brain regions are colored according to their connection weight in the connectome. (B) The local dynamics of each brain region are simulated by recurrently connected pools of excitatory (E, red circle) and inhibitory (I, blue circle) neuronal populations. In turn, brain regions are connected through the E pool, such that the excitatory inputs from other regions are weighted according to their respective connection weight, and their sum is scaled by the Global Coupling parameter *G*. The connection from the I pool to the E one—*J*_*n*_, the local inhibitory feedback—compensates for the excess of excitatory activity injected from other regions. (C) The simulated firing rate of each excitatory pool is used to generate BOLD-like signals for each brain region using the BK model. (D) Maximum memory used by the current implementation (DMF) and by ours (FastDMF) to simulate BOLD signals of different lengths. Both implementations used the connectome shown in A. The MATLAB wrapper for the FastDMF was used to make a fair comparison against the DMF MATLAB library. (E) Same as D, but for the average execution time among 10 repetitions. A Linux laptop with an Intel Core i7 8550U processor (4 cores) and 16 GB was used for the simulations.

One of the most prominent biophysically grounded whole-brain models is the Dynamic Mean Field (DMF) model (Deco et al., 2013, 2014), which results from applying a mean-field approach to single neuron models, allowing to model the dynamics of the mean firing rate of a macroscopic brain region (Amit & Brunel, 1997; van Vreeswijk & Sompolinsky, 1996) and to connect many of these regions via a connectivity matrix (Figure 1A). This approach allows us to model the local dynamics of each brain region as two interacting neural populations: one excitatory (E) and another inhibitory (I) (see Figure 1B). In addition to interacting with its coupled I pool, the E pool receives/sends excitatory inputs/outputs from/to the E pools of other regions according to the connectome blueprint, which determines the weight of this interregional interaction (Figure 1A). Finally, the simulated firing activity of each E pool is transformed into blood oxygen level-dependent (BOLD) signals using the Balloon-Windkessel (BK) model (Stephan, Weiskopf, Drysdale, Robinson, & Friston, 2007) (Figure 1C), allowing a direct comparison with empirical fMRI data. The DMF has been used to simulate BOLD signals measured during an ample range of different conditions and global brain states (Deco, Cruzat, et al., 2018; Demirtaş et al., 2019; Gatica et al., 2022; Herzog et al., 2023; Kringelbach et al., 2020; Luppi, Mediano, et al., 2022; Wang et al., 2019).

Despite these achievements, a few limitations prevent the DMF model from being more widely exploited by the scientific community. The main limitation is that current publicly available implementations of the DMF model are computationally expensive, with demanding time and memory requirements. This problem is exacerbated by two further aspects of the DMF: (a) the need to calibrate the model’s feedback inhibition control (FIC) parameters, which stabilize the firing rates of E pools and have been shown to provide more realistic activity (Deco et al., 2014) and richer dynamics (Hellyer, Jachs, Clopath, & Leech, 2016), and (b) the need to optimize the model’s free parameters to fit the statistical features of a given empirical dataset. Both aspects involve running a large number of long simulations and thus inflate the overall computational costs, which often make the requirements of the DMF prohibitive for researchers with no access to high-performance computing infrastructure. This represents an undue obstacle to the ability of neuroscientists across the globe to contribute to brain modeling research: The choice of an appropriate computational model should depend only on its suitability to address specific research questions rather than being contingent on the availability of high-performance resources.

Moreover, the high computational burden of the current implementations of the DMF not only restricts its usage but also severely restricts the spatial resolution of what can be simulated. The existent implementations of the DMF are based on parcellations with less than 100 regions—typically given by the Automatic Anatomical Labeling (AAL90) (Tzourio-Mazoyer et al., 2002) or the Desikan-Killiany (Desikan et al., 2006) parcellation—because, otherwise, it would be unfeasible to simulate brain activity based on fine-grained parcellations. However, recent advances in neuroimaging data analysis involve measuring brain activity at multiple spatial scales, from coarse to fine-grained, revealing new insights about underlying brain dynamics (Deco & Kringelbach, 2020) and its relevant operational scales (Kobeleva, López-González, Kringelbach, & Deco, 2021). Therefore, being restricted to simulating biophysically realistic brain activity at a coarse-grained spatial scale represents another barrier that hinders the ability of neuroscientists to leverage the full potential of DMF modeling.

To address both of these limitations, here, we present *FastDMF*: a time- and memory-efficient implementation of the DMF model, which reduces its computational demands so it can be run and fit efficiently on any contemporary desktop computer, dramatically widening access to this kind of computational modeling approach. The FastDMF implementation is built by leveraging several key advances:

- It provides an improved implementation of the DMF model, which is significantly faster and reduces memory consumption by several orders of magnitude.
- It uses a novel connectome-dependent local inhibitory feedback mechanism, which replaces the standard FIC optimization problem and radically reduces the number of FIC calibration parameters.
- It leverages a Bayesian optimization algorithm, which substantially reduces the number of simulations required to fit the model to empirical data (see Wischnewski, Eickhoff, Jirsa, & Popovych, 2022 for comparison with other methods). This algorithm performs a smart sampling of the parameter space instead of the grid search used in previous approaches (Deco, Cruzat, et al., 2018; Luppi, Mediano, et al., 2022).

These advances make FastDMF a computationally efficient, easily accessible, and biophysically grounded whole-brain model. The FastDMF is coded in C++ and is usable on both MATLAB and Python; all codes that implement FastDMF can be found on the public repository (gitlab.com/concog/fastdmf).

To showcase the advantages of the FastDMF, we first compare our implementation against the current implementation of the DMF model (published with Deco, Cruzat, et al., 2018) in terms of computational costs. Then, we show analytical and numerical evidence supporting a connectome-dependent linear solution to the FIC optimization problem. Finally, following advances in optimization and validation of whole-brain models (Hadida, Sotiropoulos, Abeysuriya, Woolrich, & Jbabdi, 2018; Wischnewski et al., 2022), we use Bayesian optimization to fit a resting-state fMRI recording dataset parcellated at two different spatial scales (*N* = 90 and *N* = 1,000 regions). Using less than 350 optimization iterations, which can be performed on personal computers in a matter of minutes, we achieve an excellent agreement between empirical and simulated data for both scales, showing the performance and broad applicability of the FastDMF to different scenarios. Furthermore, we leveraged the FastDMF capability to model the turbulent-like dynamics (Deco & Kringelbach, 2020) in a large cohort of 1,000 healthy participants from the Human Connectome Project (HCP). Crucially, the turbulent-like measures are defined in fine brain parcellations—typically, *N* = 1,000—and so far, the modeling of these brain properties has only been done via phenomenological models (Escrichs et al., 2022; Perl, Mininni, Tagliazucchi, Kringelbach, & Deco, 2023). The FastDMF allows us, for the first time, to run biophysically grounded simulations of turbulent-like dynamics in brain activity, which allowed us to hypothesize about the mechanisms that give rise to these dynamics: a complex interaction between long-range excitatory connections and the local E/I balance.

## RESULTS

### Fast and Efficient Computational Implementation

The first advancement to make the FastDMF widely usable is to provide an open-source implementation with two main advantages: faster execution time and less memory usage. This is achieved through three main improvements: (a) The core of FastDMF is written in C++ and takes advantage of the advanced linear algebra library Eigen (Guennebaud et al., 2010), which provides a fast and cross-platform toolkit for numerical operations. (b) The FastDMF and the BK hemodynamic function to compute the BOLD signals run in parallel, further reducing the overall execution time of the simulation. (c) The DMF and the BK numerical integrators access a shared memory via a simplified producer-consumer architecture, which, given the large difference in timescale between the BOLD and the firing rate signals, allows FastDMF to radically reduce memory usage through a shared finite-size buffer. In other words, the DMF equations are used to simulate firing rates, which are temporarily stored in a buffer and then consumed by the BK integrator to generate the slow BOLD signals. Once a batch of firing rates has been consumed by the BK integrator, the DMF integrator is notified and allowed to write on the same memory address, and the cycle begins again. This mechanism makes the total memory usage of the simulation grow with the size of the resulting BOLD signals, as opposed to the firing rate signals, which are approximately 1,000 times larger in memory.

To benchmark our implementation, we ran simulations of varying lengths using the AAL90 structural connectome (Tzourio-Mazoyer et al., 2002) and measured both memory usage and execution time. We used the MATLAB wrapper of the FastDMF to make the results comparable with the previous DMF MATLAB implementation. As expected, FastDMF was faster and more memory-efficient than the public MATLAB implementation (Deco, Cruzat, et al., 2018). Thanks to the circular buffer, memory is effectively constant for all simulation times (Figure 1D) and runtime per second of simulated activity is less than half of the MATLAB DMF (Figure 1E).

Finally, in addition to the underlying C++ implementation, the FastDMF library incorporates interfaces for both MATLAB and Python, using the Mex (MathWorks, 1999) and Boost Python (Abrahams & Grosse-Kunstleve, 2003) libraries, respectively. These enable researchers to run and analyze simulations from a language of their choice, thus making the integration of FastDMF in their existing pipelines easier.

### Linear Solution to the FIC Optimization Problem

The second main improvement was to provide a linear solution to the optimization of the FIC (Deco et al., 2014). In brief, the Global Coupling (*G*) parameter of the DMF scales the inputs that each region receives from the rest of the network, allowing to tune the model closer/farther to an optimal working point, where some desired statistical feature of the empirical neuroimaging data is reproduced, such as the Functional Connectivity (FC) or the Functional Connectivity Dynamics (FCD). To compensate for the excitation that each pool receives from the other regions via the connectome, a FIC parameter is optimized through recursive adjustments to clamp the firing rate within a neurobiologically plausible range of 3–4 Hz for each local excitatory neural population, preventing the system from entering a hyperexcitation regime. The solution to this problem, based on an iterative process of increasing and decreasing the local inhibition until convergence, is explained in detail in Deco et al. (2014), and it is implemented in the scripts accompanying the publication of Deco, Cruzat, et al. (2018) and gives the optimal local inhibitory feedback *N*-dimensional vector (*J*^opt^) for a given *G*, where *N* is the number of regions. However, for a standard setup, these scripts took more than 2 months on a 32-core cluster to converge. Even worse, running the model using different connectivity matrices would require to separately optimize the FIC for each one of them, severely hindering the endeavor of investigating how structure shapes function through biophysical DMF modeling.

Despite the lack of prior information regarding the expected *J*^opt^ in the DMF models, results from other types of whole-brain models suggest that *J*^opt^ is correlated with local connectivity measures of the structural connectivity such as the structural connectivity strength (Hellyer et al., 2016). Accordingly, here, we show that a first-order analytical solution for a stationary state where the expected values of firing rates are fixed to 3.4 Hz (average excitatory firing rate for *G* = 0) predicts that *J*^opt^ is region-specific, and its magnitude is proportional to *G* and to the local connectivity strength *β*_*n*_ ≔ ∑_*p*_ *C*_*np*_ that is the strength of node *n* computed from the anatomical connectivity matrix *C*. We divide it by 2 to compensate the double counting of the symmetric entries of the anatomical connectivity matrix (see the Materials and Methods section).

We write this solution as follows:

$$J_{n}^{opt}=\alpha \;G\;\beta_{n}+1,$$ (1)

where *α* is a scaling factor that represents the global excitation-to-inhibition (E/I) ratio (see the Materials and Methods section). According to this approximation, for a fixed *G*, *α* is the only parameter to estimate to solve the FIC problem.

**Figure 2.**
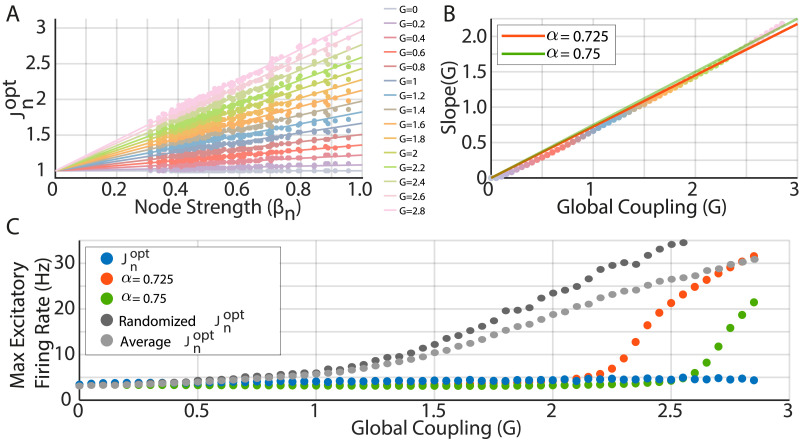
The optimal local inhibitory feedback depends on Global Coupling and local anatomical connectivity strength. (A) The optimal local inhibitory feedback ($J_{n}^{\text{opt}}$) is well approximated by a linear function of region strength (*β*_*n*_) for a wide range of *G* values (colors). (B) The slope of the $J_{n}^{\text{opt}}$ versus *β*_*n*_ scales linearly with the Global Coupling, giving an optimal slope of 0.725 (red solid line); however, using 0.75 (green solid line) better matches the high *G* values. (C) $J_{n}^{\text{opt}}$ attains stability for all *G* values between [0, 3], while the linear approximation using *α* = 0.725 diverges to the high excitability regime close to *G* = 2 (red dots). Using *α* = 0.75 (green dots) attains stability in a larger range of *G* values, diverging at *G* ∼ 2.5. To illustrate the topographical specificity of $J_{n}^{\text{opt}}$, we used a randomized version of $J_{n}^{\text{opt}}$ (dark gray circles) and a homogeneous *J*_*n*_ equal to the average $J_{n}^{\text{opt}}$ (light gray circles); both fail to control the global dynamics for increasing values of *G*.

As a previous well-known and publicly available implementation of the DMF model (Deco, Cruzat, et al., 2018) used the AAL90 parcellation, we numerically tested our first-order approximation using this parcellation. After the optimization converged, for each *G*, we plotted $J_{n}^{opt}$ versus the local connectivity strength (*β*_*n*_), finding a linear relationship whose slope linearly increased with *G* (Figure 2A, B). Then, to estimate *α* of Equation 1, we found the scaling factor between the slope and *G*, with the constraint that the slope for *G* = 0 should be 0. We used least squares to find the optimal value of *α* (0.725). However, this value gives a lower goodness of fit for higher *G* values, which are usually the values where the model better fits empirical data (Deco, Cruzat, et al., 2018; Deco et al., 2014). Accordingly, we used weighted least squares, giving 10 times more weight to *G* values larger than 2.1 (close to bifurcation with *α* = 0.725), finding an optimal value for *α* = 0.75. This approach, as expected, better matches the slope values for high *G* values and also extends the range where stability can be attained by the linear approximation (Figure 2C, green dots).

Note that a second-order approximation can be also used for the relationship between $J_{n}^{opt}$, *G*, and *β*_*n*_, but our numerical simulations show that the range where stability is attained by second-order approximations is reduced, in comparison to the first-order one.

Finally, as a control, we used two modified versions of *J*^*opt*^ to run the DMF model: (a) a randomized (shuffled) version of *J*^*opt*^ and (b) a homogeneous version, where *J*_*n*_ for all regions is equal to the average of *J*^*opt*^ (Figure 2C). We found that both alternative versions of *J*^*opt*^ fail to attain stability for high values of *G* > 0.5, showing that *J*^*opt*^ is region-specific.

Our results alleviate the huge amount of simulations required to properly tune the FIC, reducing the number of free parameters of the optimization problem from *N* to 1. In addition, we provide a biophysical interpretation of FIC in terms of the local connectivity strength and the global E/I balance.

### Reproducing the FCD of Resting-State fMRI Data for Two Different Spatial Resolutions

The third major improvement was to implement a Bayesian optimization algorithm to find the optimal set of parameters that reproduces a statistical feature of the empirical data (or more than one). The current DMF implementation finds the optimal working point by using a suboptimal strategy based on grid search. Bayesian optimization algorithms, on the other hand, estimate the objective function by sampling the parameter space efficiently, finding the minimum of a wide variety of functions (Shahriari, Swersky, Wang, Adams, & de Freitas, 2016; Ulmasov, Baroukh, Chachuat, Deisenroth, & Misener, 2016). Here, following previous applications of the DMF to fMRI data (Deco, Cruzat, et al., 2018), we chose as an objective function the Kolmogorov–Smirnov (K-S) distance between the pooled empirical FCD distribution (*FCD*_*emp*_; computed using the FCD from all subjects) and the FCD histogram obtained with the DMF (*FCD*_*DMF*_; Hansen, Battaglia, Spiegler, Deco, & Jirsa, 2015). In addition, instead of first optimizing FIC to clamp the firing rates, and then optimizing *G* to reproduce the FCD, we jointly optimized *G* and *α*, expecting that the optimal working point also satisfies the firing rate constraints. We ran the optimization until convergence to K-S distances was comparable with previous studies (Deco, Cruzat, et al., 2018; Luppi, Mediano, et al., 2022).

To exhibit the advantages of the FastDMF, in addition to the AAL90 parcellation used up to this point for consistency with previous work, we also obtained a different estimate of the healthy human connectome, based on high-resolution diffusion data (Van Essen et al., 2013) parcellated according to the most fine-grained scale of the Schaefer atlas, which comprises 1,000 functionally defined cortical regions (see the Materials and Methods section) (Schaefer et al., 2018). We also used the same parcellation to obtain the functional MRI time series, obtaining datasets in two different spatial scales.

We emphasize that the two connectomes employed for the following analyses were obtained from different groups of healthy individuals, with different acquisition parameters, different reconstruction methods and softwares, different atlases (anatomically versus functionally defined), and different resolutions (differing by one order of magnitude) and only one of which includes subcortical regions (AAL90). In other words, we chose to use two healthy connectomes that differ on virtually every relevant methodological dimension to establish the general validity of our results.

**Figure 3.**
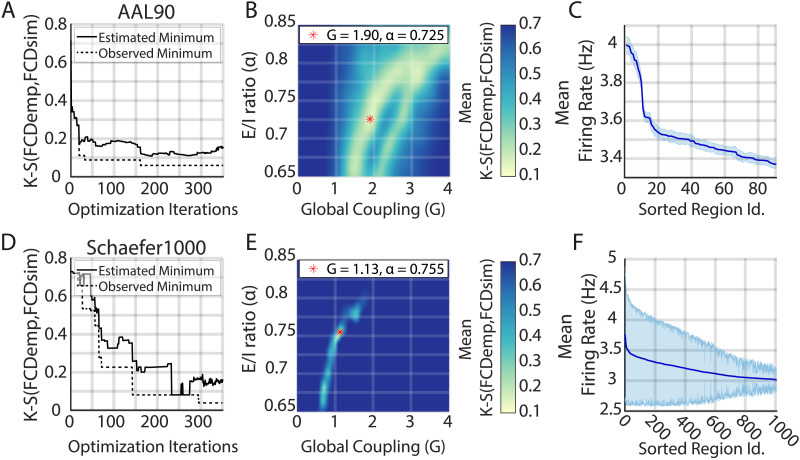
Fitting the FastDMF model to empirical data with Bayesian optimization. (A, D) Convergence of the Bayesian optimization estimates of the minimum K-S distance (solid line), for the AAL90 and the Schaefer1000 parcellation, respectively. The solid line represents the minimum of the estimated objective function by the Bayesian optimization, while the dashed line is the minimum observed across all the different initial conditions used by the optimization. (B, E) Bayesian optimization estimate of the mean K-S distance between the pooled empirical FCD (*FCD*_*emp*_) and the simulated FCD (*FCD*_*sim*_) as a function of *G* and *α*. The red asterisk shows the optimal parameters. (C, F) Simulated brain regions sorted according to their average excitatory firing rate (solid line) with their respective standard deviation computed from 96 simulations (shaded area). All regions are within the expected range of firing rates.

For both parcellations, we required less than 350 iterations to converge (Figure 3A, D); however, for the AAL90, the values stabilized around 150 iterations, while for the Schaefer1000, they stabilized around 300 iterations. These results suggest that the number of iterations required to converge could grow with the parcellation size. Note that the estimated and the observed minimum differ, as the former is the minimum of the estimated objective function (shown in Figure 3B, E, which is updated after each simulation), while the observed minimum is just one initial condition that yielded that result. As we are interested on the average behavior of the FastDMF rather than the behavior of a single initial condition, we guided our decisions by the convergence of the estimated minimum rather than of the observed one, which is shown for completeness. Note that without this optimization approach, we would have to simulate a subset of parameter combinations several times, taking the risk of using a too-coarse binning of the parameters space (to save time), thus missing the minimum. In addition, the Bayesian optimization algorithm can take advantage of parallel computing, making the optimization procedure even faster.

Indeed, we found comparable values of goodness of fit for the two spatial scales (K-S ≈ 0.15; Figure 3A, D). Note that a difference in the optimal *G* value between the two spatial scales is to be expected: not just because two different connectomes were used but also, mathematically, because of the larger number of regions in the Schaefer1000, which in consequence, increases the average *β*_*n*_, reducing the magnitude of the Global Coupling required to drive the system to a high-excitability regimen.

Crucially, for both connectomes, *α* is within the values shown in Figure 2, demonstrating the robustness and generalizability of our estimation of this parameter, even when the empirical healthy connectomes used for the simulation originate from different cohorts and were obtained through different reconstruction approaches at different resolutions.

**Figure 4.**
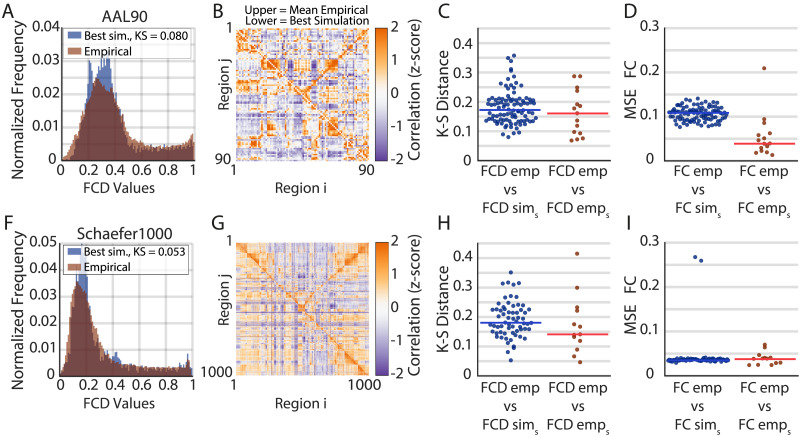
Comparison between empirical and simulated FCD and FC for two spatial scales. (A, F) FCD distribution for the pooled empirical data (red) and for the best simulation (blue) for the AAL90 and the Schaefer1000 parcellations, respectively. (B, G) FC matrix for the average empirical (upper triangular) and for the best simulation (lower triangular). (C, H) K-S distance between simulated *FCD*_*sim*_*s*__ and pooled empirical *FCD*_*emp*_ (blue). The K-S distance between single subjects, *FCD*_*emp*_*s*__, and pooled empirical, *FCD*_*emp*_, is shown in red. The horizontal line is the median. (D, I) Same as C and H, but for mean squared error (MSE) between FC matrices. In this case, *FC*_*emp*_ is the average FC between all subjects.

We further evaluated the Bayesian optimization results by using the optimal parameters to run 96 simulations with different initial conditions and a simulation length comparable with resting-state recordings (∼10 min). First, we checked the average excitatory firing rates, finding that for both spatial scales, they remained in the desired range (3–4 Hz; Figure 3C, F). However, given the increased number of regions of the Schaefer1000 parcellation, the average firing rates showed increased variance, reflecting more susceptibility to the initial conditions. Second, we checked that the best simulation closely matched the empirical FCD distribution (Figure 4A, F), reproducing the bimodality of the empirical FCD distribution. Note that the resting-state FC matrix is also well captured (Figure 4B, G), even when it was not included as an objective function in the fitting procedure.

Finally, we aimed to evaluate the goodness of fit of the simulated FC and FCD with respect to the variability of the empirical population (Figure 4C, D, H, I). For example, if we take the FC of a single subject *s* (*FC*_*emp*_*s*__), how similar is this subject to the average of the population *FC*_*emp*_? Then, we can make the same question for one simulation *s* (*FC*_*sim*_*s*__) with respect to the same population average *FC*_*emp*_, providing a notion of goodness of fit that takes into account the empirical population variability. We did this procedure for both parcellations and for the FCD (Figure 4C, H; using the KS distance between FCD histograms) and the FC (Figure 4D, I; using the mean squared error between FCs). For the FCD and the AAL90 parcellation, we found no significant difference (Figure 4C; Wilcoxon rank test *p* > 0.05), while for the Schaefer1000, we found small but significant differences (Figure 4H; *p* < 0.05). In the case of the FC, we found significant differences for the AAL90 (Figure 4D, *p* < 0.05) but no significant differences for the Schaefer1000 (Figure 4I; *p* > 0.05). This results show that the optimal parameters can generate simulations whose FCD and FC are similar to the ones obtained from sampling the empirical population. As may be expected from optimized simulations, they can exhibit small but significant differences.

We highlight that this approach represents a considerable reduction in the number of simulations required to fit the model to empirical data, even more for the Schaefer1000 parcellation, which is prohibitive in current implementations. In addition, we remark that the optimal set of parameters that reproduces the empirical FCD also satisfies the local stability condition (i.e., stable firing rates) and also captures important structures of the FC matrix, showing the degree of generalization of the FastDMF model.

### Generation of Brain Turbulent-Like Dynamics

Finally, we used the FastDMF to explore its ability to generate turbulent-like dynamics, a phenomenon observed in human fMRI recordings parcellated at fine-grained scales (*N* = 1,000) (Deco & Kringelbach, 2020). In fluids, turbulent dynamics facilitate optimal energy transfer via scale-free mixing, which has inspired its application to brain dynamics (Deco & Kringelbach, 2020; Perl et al., 2023). In the brain, the quantification of turbulent-like dynamics provides useful distinctions and characterizations of different brain states (Escrichs et al., 2022). Building upon previous results of the application of turbulence theory to brain dynamics, here, we aimed to use the FastDMF to provide potential biophysical insights for the generation of brain turbulent-like dynamics.

**Figure 5.**
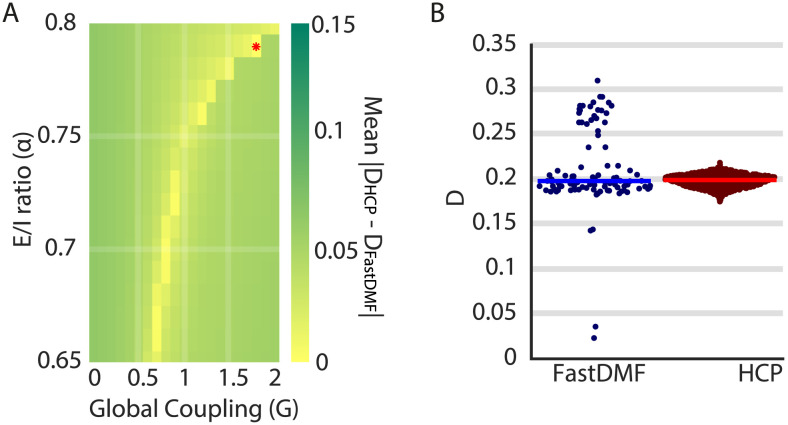
FastDMF reproduces brain turbulent-like dynamics. (A) The mean absolute difference of the level of turbulent-like dynamics, *D*, for the HCP and data simulated with the FastDMF as a function of *α* and *G* parameters of the FastDMF. The red asterisk denotes the best fit. (B) Distribution of turbulence amplitude for the best fit of the FastDMF (blue dots; red asterisk of panel A) and of the HCP (red dots). Each dot corresponds to a different initial condition (*N* = 96) and subject (*N* = 1,000), respectively. Horizontal lines denote the median of the distribution. No significant differences were found (Wilcoxon rank sum test, *p* value = 1).

We used previous analysis of brain turbulent-like dynamics applied to the HCP (parcellated with the Schaefer1000 parcellation) and explored the relationship between *α* and *G* to generate these dynamics. Here, we used the metric *D* as a proxy for the amplitude of turbulent like-dynamics (see the Materials and Methods for details on the computation; Equation 11). For the FastDMF, we estimated *D* in a grid of *α* (0.65–0.8) and *G* (0–2) values and looked for the best fit to the empirical *D* value observed in the HCP dataset. Our results show that the best combination between *α* and *G* (red asterisk on Figure 5A) generates the same level of turbulent-like brain dynamics as the empirical (Figure 5B; Wilcoxon rank sum test *p* value = 1). Beyond the best fit, results show that there is a nonlinear interaction between *G* and *α* that generates a whole region where human-like turbulent dynamics emerge. This empirical observation suggests a mechanistic explanation for turbulence-like dynamics: Turbulent-like brain dynamics emerge from a complex interaction between excitatory long-range connectivity and local E/I balance. Notably, we found that this region in parameter space co-localizes with the region comprised by the 10% best fits the FCD obtained with a completely different dataset (Supporting Information Figure S1). This may imply that regardless of the dataset, the FastDMF generates dynamics as rich as the human brain using the same combinations of *α* and *G*, highlighting the generalization potential of the model.

Thus, we demonstrated the generalization ability of the FastDMF and its potential to provide biophysical mechanistic insights underlying the generation of turbulent-like dynamics. The advancements of the FastDMF allow this kind of exploration for fine-grained parcellations within reasonable computational constraints.

## DISCUSSION

In this paper, we introduced the FastDMF: an accessible, efficient, and biophysically grounded whole-brain model. Thanks to a combination of optimized implementation, efficient parameter search, and a novel method of estimating the FIC, the FastDMF can fit statistical aspects of empirical fMRI data using parcellations ranging from tenths (e.g., the AAL90; Tzourio-Mazoyer et al., 2002) to thousands (e.g., the Schaefer1000; Schaefer et al., 2018) of brain regions, highlighting the flexibility of our approach and its robustness to a variety of methodological choices. The FastDMF allows, for first time, to run biophysically grounded whole-brain simulations accounting for brain dynamics that are only observed in fine parcellations, such as turbulence-like dynamics, which opens the way to a range of novel analysis with the potential to substantially deepen our understanding of brain dynamics and evoked responses (Deco & Kringelbach, 2020; Deco et al., 2021; De Filippi et al., 2021; Escrichs et al., 2022).

Our proof of concept on studying the brain dynamics on fine-grained parcellations suggests that both the rich repertoire of empirical FCD and turbulent-like dynamics are generated by a nonlinear interaction between long-range excitatory connections and the local E/I balance. Note that the dataset used for fitting the FCD and turbulence were different (data from Tagliazucchi, Carhart-Harris, Leech, Nutt, & Chialvo, 2014 and HCP, respectively; see the Participants section), which highlights the generalizability potential of the FastDMF to different fMRI resting-state datasets (see Supporting Information Figure S1). However, further research is needed to establish a formal relationship between the FCD, turbulent-like dynamics, global connectivity, and the E/I balance.

The presented case studies show how our method provides accurate results for a wide range of *G* coupling parameter values, thus outperforming existing tools for computing FIC using the DMF. Different alternatives have been proposed to model the FIC. A plasticity rule for a time-varying FIC has been used in a modified version of the DMF (Naskar et al., 2021), avoiding the optimization process, at the expense of increased model complexity. It has been suggested that the optimal FIC obtained by the iterative process of Deco et al. (2014) corresponds to the integrated form of the time-varying FIC (Hellyer et al., 2016). Moreover, despite the increased model complexity, the inclusion of a plasticity rule into the DMF could enhance the dynamic range and stability. For example, for high-coupled regimes, the static FIC cannot achieve stability, while a dynamic FIC could better track sudden increases in excitation. We believe that for the high-coupled regime, a dynamic homeostatic mechanism for the FIC could produce a slow wave-like activity, as sudden increases in the excitation could be compensated by sudden increases in inhibition, thus producing an oscillation between high and low activity. While our main purpose was to show the use of the FastDMF rather than explaining the possible mechanisms involved in the FIC or their potential functional implications, our model provides novel evidence in favor of a connection between the strength of the nodes in the connectome and FIC.

Needless to say, there is still room for further improvement of the FastDMF. More general parameters allowing the incorporation of multiple receptor maps to neuromodulate the activity of each brain region, principal gradient of gene expression, or anatomical measures could be implemented in future developments (Kong et al., 2021). Another direction for future research would be to explore the performance of our FastDMF in the spatial limits of parcellations; the cortical surface gray matter can be used as nodes, with higher spatial resolution than parcellated brain regions. Additionally, new data modalities that can be derived from firing rates such as local field potentials or EEG-like periodic and aperiodic activity could be future avenues of development.

The study of whole brain activity is an exciting research avenue (Deco & Kringelbach, 2020; Einevoll et al., 2019; Munn et al., 2021) that needs to be better developed to understand the causal interplay between brain structure and function in different brain health conditions, addressing the specific relevance of factors such as neuronal dynamics, neurotransmitter receptor density, and anatomical connectivity, among others. This, in turn, might accelerate brain research, identifying the biophysical mechanistic principles that relate different levels of brain organization, opening in this way the road for the development of new treatments to prevent and cure brain disease (Cofré et al., 2020; Deco & Kringelbach, 2014; Luppi, Mediano, et al., 2022). In line with this perspective, the FastDMF provides a general and accessible tool of simulation and analysis to be applied in multiple neuroimaging scenarios that can be called from Python and MATLAB, which should favor its use by the scientific community.

## MATERIALS AND METHODS

### DMF Model

The DMF model introduced by Deco and colleagues (Deco, Cruzat, et al., 2018; Deco et al., 2014) uses a set of coupled stochastic differential equations to model the dynamics of the activity at one or more interacting brain regions. In this model, each brain region is modeled as two coupled neuronal masses—one excitatory and one inhibitory—and considers excitatory and inhibitory synaptic currents mediated by NMDA and GABA_A_ receptors, respectively. Different brain regions (usually defined by a given brain parcellation) are coupled via their excitatory populations exclusively, according to the structural connectivity matrix.

The key idea behind the mean-field approximation is to reduce a high-dimensional system of randomly interacting elements to a system of elements treated as independent. This approach represents the average activity of a homogeneous population of neurons by the activity of a single unit of this class.

The model establishes that the *n*-th brain area obeys the following equations:

$$I_{n}^{\left(\text{E}\right)}=W_{\text{E}}I_{0}+w_{+}J_{\text{NMDA}}S_{n}^{\left(\text{E}\right)}+{GJ}_{\text{NMDA}}\sum_{p}^{N}C_{np}S_{p}^{\left(\text{E}\right)}-J_{n}^{\text{FIC}}S_{n}^{\left(\text{I}\right)},$$ (2A)

$$I_{n}^{\left(\text{I}\right)}=W_{\text{I}}I_{0}+J_{\text{NMDA}}S_{n}^{\left(\text{E}\right)}-S_{n}^{\left(\text{I}\right)},$$ (2B)

$$r_{n}^{\left(\text{E}\right)}=F_{\text{E}}\left(I_{n}^{\left(\text{E}\right)}\right)=\frac{g_{\text{E}}\left(I_{n}^{\left(\text{E}\right)}-I_{thr}^{\left(\text{E}\right)}\right)}{1-\exp\left(-d_{\text{E}}\;g_{\text{E}}\left(I_{n}^{\left(\text{E}\right)}-I_{thr}^{\left(\text{E}\right)}\right)\right)},$$ (2C)

$$r_{n}^{\left(\text{I}\right)}=F_{\text{I}}\left(I_{n}^{\left(\text{I}\right)}\right)=\frac{g_{\text{I}}\left(I_{n}^{\left(\text{I}\right)}-I_{thr}^{\left(\text{I}\right)}\right)}{1-\exp\left(-d_{\text{I}}\;g_{\text{I}}\left(I_{n}^{\left(\text{I}\right)}-I_{thr}^{\left(\text{I}\right)}\right)\right)},$$ (2D)

$$\frac{{dS}_{n}^{\left(\text{E}\right)}\left(t\right)}{dt}=-\frac{S_{n}^{\left(\text{E}\right)}}{\tau_{\text{NMDA}}}+\left(1-S_{n}^{\left(\text{E}\right)}\right)\gamma r_{n}^{\left(\text{E}\right)}+\sigma v_{n}\left(t\right),$$ (2E)

$$\frac{{dS}_{n}^{\left(\text{I}\right)}\left(t\right)}{dt}=-\frac{S_{n}^{\left(\text{I}\right)}}{\tau_{\text{GABA}}}+r_{n}^{\left(\text{I}\right)}+\sigma v_{n}\left(t\right)$$ (2F)

Above, for each excitatory (E) and inhibitory (I) neural mass, the quantities *I*_*n*_, *r*_*n*_, and *S*_*n*_ represent its total input current, firing rate, and synaptic gating variable, respectively. The functions *F*_E_(·), *F*_I_(·) determine the transfer functions (characterized by an *F-I curve*), representing the nonlinear relationship between the input current and the output firing rate of excitatory and inhibitory neural populations. Crucial for our derivations, $J_{n}^{\text{FIC}}$ is the local FIC of region *n*, and *v*_*n*_ is an uncorrelated Gaussian noise injected to region *n* (Wong & Wang, 2006). The remaining quantities involved in these equations are introduced in Table 1. The interested reader is referred to the original publication for further details (Deco et al., 2014)

**Table 1.** DMF model parameters

*Parameter*	*Symbol*	*Value*
External current	*I*_0_	0.382 nA
Excitatory scaling factor for *I*_0_	*W*_*E*_	1
Inhibitory scaling factor for *I*_0_	*W*_*I*_	0.7
Local excitatory recurrence	*w*_+_	1.4
Excitatory synaptic coupling	*J*_*NMDA*_	0.15 nA
Threshold for *F*($I_{n}^{\left(\text{E}\right)}$)	$I_{thr}^{\left(\text{E}\right)}$	0.403 nA
Threshold for *F*($I_{n}^{\left(\text{I}\right)}$)	$I_{thr}^{\left(\text{I}\right)}$	0.288 nA
Gain factor of *F*($I_{n}^{\left(\text{E}\right)}$)	*g*_*E*_	310 nC^−1^
Gain factor of *F*($I_{n}^{\left(\text{I}\right)}$)	*g*_*I*_	615 nC^−1^
Shape of *F*($I_{n}^{\left(\text{E}\right)}$) around $I_{thr}^{\left(\text{E}\right)}$	*d*_*E*_	0.16 s
Shape of *F*($I_{n}^{\left(\text{I}\right)}$) around $I_{thr}^{\left(\text{I}\right)}$	*d*_*I*_	0.087 s
Excitatory kinetic parameter	*γ*	0.641
Amplitude of uncorrelated Gaussian noise *v*_*n*_	*σ*	0.01 nA
Time constant of NMDA	*τ*_*NMDA*_	100 ms
Time constant of GABA	*τ*_*GABA*_	10 ms

NMDA = N-methyl-D-aspartic acid; GABA-A = gamma aminobutyric acid - A.

In all the simulations reported in this article, the above stochastic differential equations were discretized and solved using the Euler-Maruyama integration method (Kloeden & Platen, 2013) and using the parameter values shown in Table 1. The first 10 s of the simulation was discarded to ensure the stability of the dynamical system.

### BK Hemodynamic Model of BOLD Activity

To transform the simulated excitatory firing rate signals into BOLD activity, we used the BK model following Stephan et al. (2007), which defines a dynamic relationship between firing rates and BOLD signals. This model defines a vasodilatory signal *s*_*n*_ for each region, subject to autoregulatory feedback *γ*_*BW*_ and decay *κ*, which influences the blood inflow *f*_*n*_, inducing changes in blood volume *v*_*n*_, and deoxyhemoglobin content *q*_*n*_ following:

$$\frac{{ds}_{n}}{dt}=0.5r_{n}^{\left(\text{E}\right)}+3-\kappa s_{n}-\gamma_{BW}\left(f_{n}-1\right),$$ (3A)

$$\frac{{df}_{n}}{dt}=s_{n},$$ (3B)

$$\tau_{BW}\frac{{dq}_{n}}{dt}=\frac{f_{n}{\left(1-\rho \right)}^{1/f_{n}}}{\rho }-\frac{q_{n}v_{n}^{1/\alpha_{BW}}}{v_{n}},$$ (3C)

$$B_{n}=V_{0}\left[k_{1}\left(1-q_{n}\right)+k_{2}\left(1-q_{n}/v_{n}\right)+k_{3}\left(1-v_{n}\right)\right]$$ (3D)

where *B*_*n*_ is the simulated BOLD signal; *ρ* is the resting oxygen extraction fraction; *α*_*BW*_ represents the resistance of the veins; *τ*_*BW*_ is a time constant; and *k*_1_, *k*_2_, and *k*_3_ are coefficients estimated from data. Accordingly, all biophysical parameters were taken from previous work (Stephan et al., 2007).

### A First-Order FIC Approximation

Here, we provide an analytical derivation of how $J_{n}^{\text{FIC}}$ depends on other model parameters. The derivation is based on two assumptions, whose range of validity is also discussed.

As a first step in the derivation, we write down the discrete time form of $S_{n}^{\left(\text{E}\right)}$ (*t*) using Equation 2E according to the Euler-Maruyama method, which gives

$$S_{n}^{\left(\text{E}\right)}\left(t+1\right)=S_{n}^{\left(\text{E}\right)}\left(t\right)+\left(-\frac{S_{n}^{\left(\text{E}\right)}\left(t\right)}{\tau_{\text{NMDA}}}+(1-S_{n}^{\left(\text{E}\right)}\left(t\right))\gamma r_{n}^{\left(\text{E}\right)}\left(t\right)\right)dt+\sigma v_{n}\left(t\right)\sqrt{dt}.$$ (4)

Then, we calculate the steady-state average of $S_{n}^{\left(\text{E}\right)}$ by computing the expected value to Equation 4. Using the steady-state property and introducing the shorthand notation $𝔼[S_{n}^{\left(\text{E}\right)}]≔𝔼[S_{n}^{\left(\text{E}\right)}\left(t\right)]=𝔼[S_{n}^{\left(\text{E}\right)}\left(t+1\right)]$, a direct calculation shows that

$$𝔼[S_{n}^{\left(\text{E}\right)}\left(t\right)]=\gamma \;\tau_{\text{NMDA}}\left(𝔼[r_{n}^{\left(\text{E}\right)}]-𝔼[S_{n}^{\left(\text{E}\right)}\left(t\right)r_{n}^{\left(\text{E}\right)}\left(t\right)]\right).$$ (5)

To proceed further in the derivation, one needs an expression for $𝔼[S_{n}^{\left(\text{E}\right)}\left(t\right)r_{n}^{\left(\text{E}\right)}\left(t\right)]$. It can be found via numerical simulations that the covariance between these two variables is lower than 0.1, so we assume that $𝔼[S_{n}^{\left(\text{E}\right)}\left(t\right)r_{n}^{\left(\text{E}\right)}\left(t\right)]=𝔼[S_{n}^{\left(\text{E}\right)}]𝔼[r_{n}^{\left(\text{E}\right)}]$ (Assumption 1). Plugging this relationship into Equation 5 yields

$$𝔼[S_{n}^{\left(\text{E}\right)}]=\frac{\gamma \;\tau_{\text{NMDA}}𝔼[r_{n}^{\left(\text{E}\right)}]}{1+\gamma \;\tau_{\text{NMDA}}𝔼[r_{n}^{\left(\text{E}\right)}]}.$$ (6)

The FIC is assumed to successfully regulate the average excitatory firing rate if, for any *G*, they remain close to the firing rates obtained for the disconnected case (*G* = 0): $𝔼[r_{n}^{\left(\text{E}\right)}]$ = 3.4 for all the nodes of the network. Importantly, this implies that $𝔼[S_{n}^{\left(\text{E}\right)}]$ is also constant across nodes, and using Equation 6, one finds that $𝔼[S_{n}^{\left(\text{E}\right)}]$ = 0.179. Numerical simulations for uncoupled regions give a steady-state average of $S_{n}^{\left(\text{E}\right)}$ = 0.179, providing an empirical confirmation of the accuracy of this approximation for that scenario.

As a second step of the derivation, let us study the expected values of $I_{n}^{\left(\text{I}\right)}$, $r_{n}^{\left(\text{E}\right)}$, $r_{n}^{\left(\text{I}\right)}$, and $S_{n}^{\left(\text{I}\right)}$ using the equations provided in the previous section. The equations for $I_{n}^{\left(\text{I}\right)}$ and $S_{n}^{\left(\text{I}\right)}$ are linear, so computing the expected value is straightforward; in contrast, $𝔼[r_{n}^{\left(\text{E}\right)}]=𝔼[F(I_{n}^{\left(\text{E}\right)})]$ involves a nonlinear function *F*. To move forward, we adopt a first-order Taylor expansion and assume that the approximation $𝔼[r_{n}^{\left(\text{E}\right)}]\approx F(𝔼[I_{n}^{\left(\text{E}\right)})]$ is accurate (Assumption 2). By doing this, one obtains a system of four nonlinear equations with four unknowns (the expected values of $I_{n}^{\left(\text{I}\right)}$, $I_{n}^{\left(\text{E}\right)}$, $S_{n}^{\left(\text{I}\right)}$, and $r_{n}^{\left(\text{I}\right)}$), which can be solved numerically. Since the inputs to this system of equations—that is, 𝔼[*r*^(E)^] and 𝔼[*S*^(E)^]—adopt the same values for all regions, the results yield the same values for all regions.

As a final step, we apply the expected value of Equation 2A. Using the fact that $𝔼\left[S_{n}^{\left(\text{E}\right)}\right]$ does not depend on *n*, a direct calculation shows that

$$𝔼[I_{n}^{\left(\text{E}\right)}]=W_{E}I_{0}+w_{+}J_{\text{NMDA}}𝔼[S^{\left(\text{E}\right)}]+J_{\text{NMDA}}𝔼[S^{\left(\text{E}\right)}]G\beta_{n}-J_{n}^{\text{FIC}}𝔼[S^{\left(\text{I}\right)}],$$ (7)

where *β*_*n*_ ≔ ∑_*p*_ *C*_*np*_ is the strength of node *n*. To conclude, one can solve the above equation for $J_{n}^{\text{FIC}}$ to obtain

$$J_{n}^{\text{FIC}}=\alpha G\beta_{n}+c.$$ (8)

Above, *α* = $\frac{J_{\text{NMDA}}𝔼\left[S^{\left(\text{E}\right)}\right]}{𝔼\left[S^{\left(\text{I}\right)}\right]}$ is a ratio between the expected fraction of open NMDA to GABA channels, which represents a global E/I balance parameter, and *c* = $\frac{1}{𝔼\left[S^{\left(\text{I}\right)}\right]}$ (*W*_*E*_*I*_0_ + *w*_+_*J*_NMDA_ 𝔼[*S*^(E)^] − 𝔼[*I*^(E)^]) is an offset parameter that corresponds to the $J_{n}^{\text{FIC}}$ for uncoupled regions. To find values of *α* and *c*, one can plug 𝔼[*r*^(E)^] = 3.4, the aforementioned expected values obtained for uncoupled regions, and the usual model parameters, to obtain *α* = 0.67 and *c* = 0.97. To match the value of $J_{n}^{\text{FIC}}$ = 1 for uncoupled regions (corresponding to *G* = 0), one can use approximately *c* = 1.

Please note that this first-order approximation is based on the expected values obtained for uncoupled regions, so one should expect that Assumption 2 may become less accurate as *G* increases, which reflects that the nonlinear effects of *F*(·) play a more important role in determining the optimal value of $J_{n}^{\text{FIC}}$ to attain stability.

### Resting-State fMRI Signals

#### Participants.

A total of 63 healthy subjects (36 females; mean ± SD, 23 ± 43.3 years) were selected from a dataset previously described in a sleep-related study by Tagliazucchi and Laufs (Tagliazucchi et al., 2014). Participants entered the scanner at 7 PM and were asked to relax, close their eyes, and not fight the sleep onset. We selected 13 subjects during the resting-state, awake condition, with a time series of at least 200 volumes to perform our analysis. The local ethics committee approved the experimental protocol (Goethe-Universität Frankfurt, Germany, protocol number: 305/07), and written informed consent was asked to all participants before the experiment. The study was conducted according to the Helsinki Declaration on ethical research.

The dataset used in the turbulent-like modeling study is from an independent publicly accessible collection of fMRI data. We used a sample comprising 1,000 participants, drawn from the HCP’s March 2017 public data release. It can be obtained in the following link: https://humanconnectome.org/study/hcp-young-adult/overview.

#### MNI data acquisition.

MRI images were acquired on a 3-T Siemens Trio scanner (Erlangen, Germany), and fMRI acquisition parameters were 1,505 volumes of T2-weighted echo planar images, TR/TE = 2,080 ms/30 ms, matrix of 64 × 64, voxel size of 3 × 3 × 3 mm^3^, distance factor of 50%, and field of view of 192 mm^2^.

The 1,000 individuals from the HCP underwent scanning using a 3-T connectome Skyra scanner (Siemens). Our analysis involved a single resting-state fMRI acquisition lasting around 15 min, conducted on the same day. For comprehensive information on participants, acquisition protocols, and data preprocessing for both the resting state and the seven tasks, the HCP website (https://www.humanconnectome.org/) offers detailed resources.

### Brain Parcellation to Extract BOLD Time Series

#### AAL90.

To extract the time series of BOLD signals from each participant in a coarse parcellation, we used the AAL90 parcellation with 90 brain areas anatomically defined in Tzourio-Mazoyer et al. (2002).

#### Schaefer1000.

To extract the time series of BOLD signals from each participant, on a finer scale, we used the Schaefer functional parcellation with 1,000 brain areas, which was based on the estimation from a large dataset (*n* = 1,489) (Schaefer et al., 2018).

#### Filtering.

BOLD signals (empirical or simulated) were filtered with a Butterworth (Order 2) band-pass filter in the 0.01–0.1 frequency range.

### FC and FCD

The FC matrix was obtained by computing the Pearson correlation coefficient between all the pairs of simulated or recorded BOLD signals. The FCD was obtained by computing the FC(*t*), where *t* is given by consecutive sliding windows of 30 time points, 28 points of overlap. Then, we vectorized each of the FC(*t*) by taking the upper triangular and finally computed the Pearson correlation coefficient between all these vectors to obtain the FCD matrix.

### Turbulence Measures

The turbulence-like brain dynamics measures are defined in previous works (Deco & Kringelbach, 2020; Escrichs et al., 2022). In brief, these measures are based on the local Kuramoto order parameter, with modulus *R*_*n*_ and phase *ν*_*n*_ representing the local level of synchronization, computed as the spatial average of the complex phase factor of the local oscillators weighted by the coupling calculated through:

$$R_{n}^{\lambda }\left(t\right){\text{e}}^{i\nu_{n}\left(t\right)}=\sum_{p}\left[\frac{{\text{K}}_{nq}^{\lambda }}{\sum_{q}K_{nq}^{\lambda }}\right]{\text{e}}^{i\varphi_{p}\left(t\right)}$$ (9)

where *φ*_*p*_(*t*) are the phases of the spatiotemporal data obtained by the Hilbert transform and where $K_{np}^{\lambda }$ is the local weighting kernel between node *n* and *p*, and *λ* defines the scaling of this local weighting:

$$K_{nq}^{\lambda }={\text{e}}^{-\lambda r\left(n,q\right)}$$ (10)

where *r*(*n*, *q*) is the Euclidean distance between the nodes *n* and *p*. The scaling is fixed at *λ* = 0.18, computed from the empirical data as reported by Deco and Kringelbach (2020).

In particular, we focused our investigation on the level of turbulence *D*, which is defined as the standard deviation across time (*t*) and brain areas (*n*) of the modulus of the local Kuramoto order parameter (*R*^*λ*^):

$$D={\langle {\left(R^{\lambda }\right)}^{2}\rangle }_{\left(n,t\right)}-{\langle R^{\lambda }\rangle }_{\left(n,t\right)}^{2}$$ (11)

### Bayesian Optimization

A MATLAB implementation of Bayesian optimization (Shahriari et al., 2016; Ulmasov et al., 2016) with an expected improvement acquisition function was used to optimize the DMF model parameters. To find the optimal point *x* in a parameter space associated with an objective function *F*(*x*), the Bayesian optimization follows:

$$x_{n+1}=\arg\;\max_{x}\;\alpha \left(x;{𝒟}_{n}\right)$$ (12)

- *x*_*n*+1_: The next point to evaluate the objective function.
- arg max_*x*_: The argument of the maximum, meaning that we choose the *x* that maximizes the acquisition function.
- *α*(*x*; 𝒟_*n*_): The acquisition function, which guides the parameter space exploration and depends on the current surrogate model of the objective function and the data 𝒟_*n*_ observed so far. It quantifies the trade-off between exploration (sampling areas with high uncertainty) and exploitation (sampling areas where the model predicts high objective values). By maximizing the acquisition function, Bayesian optimization efficiently searches the input space to find the global optimum of the objective function with as few evaluations as possible.
- 𝒟_*n*_: The set of observations (data points) available up to iteration *n*, which includes the input points *x*_1_, *x*_2_, …, *x*_*n*_ and their corresponding function values *F*(*x*_1_), *F*(*x*_2_), …, *F*(*x*_*n*_).

The objective function was defined as the K-S distance between the histogram of the pooled empirical FCD (*FCD*_*emp*_) and the histogram of the simulated FCD (*FCD*_*DMF*_). We simulated 500 s of BOLD signals sampled at 2 s (the TR used in the empirical data), generating a number of time points comparable with the empirical BOLD signals. The optimization was run assuming a stochastic objective function, letting the algorithm to randomly select the initial conditions for each simulation.

### Structural Connectivity

#### AAL90.

The structural connectome was obtained by applying DTI to diffusion-weighted imaging recordings from 16 healthy right-handed participants (11 men and 5 women, mean age: 24.75 ± 2.54 years) recruited online at Aarhus University, Denmark, as used in previous studies (Cabral, Hugues, Kringelbach, & Deco, 2012; Deco, Cruzat, et al., 2018; Ipiña et al., 2020). Briefly, the construction of the structural connectivity matrix (SC) was performed following the following steps: The regions defined using the AAL template (Tzourio-Mazoyer et al., 2002) were warped from the MNI space to the diffusion MRI native space using the FLIRT tool from the FSL toolbox (www.fmrib.ox.ac.uk/fsl; FMRIB, Oxford). Then, probabilistic tractography with default parameters of the FSL diffusion toolbox (Fdt) was used to estimate the connections between regions. The local probability distribution of fiber direction at each voxel was estimated following Behrens et al. (2003), and the probtrackx tool in Fdt was used for the automatic estimation of crossing fibers within each voxel. Using a sampling of 5,000 streamlines per voxel, the connection probability from a seed voxel *i* to another voxel *j* was defined as the proportion of fibers flowing through voxel *i* that reach voxel *j* (Behrens, Berg, Jbabdi, Rushworth, & Woolrich, 2007). The fraction of the sampled fibers in all voxels in a region *i* that reach any voxel in region *j* in an AAL region *i* determines the connection probability *P*_*ij*_ between those regions. Due to the dependence of tractography on the seeding location, the probability *P*_*ij*_ is not necessarily equivalent to *P*_*ji*_. However, these two probabilities are highly correlated across the brain for all participants (the least Pearson *r* = 0.70, *p* < 10 − 50), and thus, the unidirectional connectivity probability *P*_*ij*_ between regions *i* and *j* was defined by averaging these two connectivity probabilities. This connectivity was considered as a measure of the structural connectivity resulting in a 90 × 90 symmetric weighted matrix *C* representing the network organization of each brain. A group-averaged structural connectivity matrix was obtained by averaging across all 16 healthy participants.

#### Schaefer1000.

As described in previous studies (Deco & Kringelbach, 2020), we used the HCP database, which contains diffusion spectrum and T2-weighted neuroimaging data. Specifically, we estimated the structural connectivity using the HCP diffusion MRI dataset provided by the Special HCP diffusion MRI, which uses excellent protocols, taking 89 min for each of 32 HCP participants at the Massachusetts General Hospital center. A detailed description of the acquisition parameters can be found on the HCP website (Setsompop et al., 2013). The precise preprocessing is described in detail in Horn and colleagues (2017) In brief, the data were processed by using a q-sampling imaging algorithm implemented in DSI Studio (https://dsi-studio.labsolver.org). A white matter mask was computed by segmenting the T2-weighted images and co-registering the images to the b0 image of the diffusion data using SPM12. A total of 200,000 fibers were sampled within the white matter mask for each HCP participant. Fibers were transformed into MNI space using Lead-DBS (Horn & Blankenburg, 2016). Finally, we used the standardized methods in Lead-DBS to extract the structural connectomes from the Schaefer1000 parcellation (Schaefer et al., 2018). A group-averaged structural connectivity matrix was obtained by averaging across all 32 healthy participants.

## ACKNOWLEDGMENTS

R.H. was funded by CONICYT scholarship CONICYT-PFCHA/Doctorado Nacional/2018-21180428. P.M. is funded by the Wellcome Trust (Grant No. 210920/Z/18/Z). F.R. is supported by the Ad Astra Chandaria Foundation. A.I.L. is funded by a Gates Cambridge Scholarship (OPP 1144). E.T. is funded by Agencia Nacional De Promocion Cientifica Y Tecnologica (Argentina) (Grant No. PICT-2018-03103). R.C. was supported by the Human Brain Project, H2020-945539. Y.S.-P. was supported by the project NEurological MEchanismS of Injury, and Sleep-like cellular dynamics (NEMESIS) (ref. 431 101071900) funded by the EU ERC Synergy Horizon Europe. G.D. was supported by the project NEurological MEchanismS of Injury, and Sleep-like cellular dynamics (NEMESIS) (ref. 101071900) funded by the EU ERC Synergy Horizon Europe, by the NODYN Project PID2022-136216NB-I00 financed by the MCIN/AEI/10.13039/501100011033/FEDER, UE, the Ministry of Science and Innovation, the State Research Agency, and the European Regional Development Fund; by the AGAUR research support grant (ref. 2021 SGR 00917) funded by the Department of Research and Universities of the Generalitat of Catalunya; and by the project eBRAIN-Health - Actionable Multilevel Health Data (id 101058516), funded by the EU Horizon Europe.

## SUPPORTING INFORMATION

Supporting information for this article is available at https://doi.org/10.1162/netn_a_00410.

## AUTHOR CONTRIBUTIONS

Rubén Herzog: Conceptualization; Data curation; Formal analysis; Investigation; Methodology; Software; Visualization; Writing – original draft; Writing – review & editing. Pedro Mediano: Formal analysis; Methodology; Software; Writing – original draft; Writing – review & editing. Fernando Rosas: Conceptualization; Supervision; Writing – original draft; Writing – review & editing. Andrea I. Luppi: Supervision; Writing – original draft; Writing – review & editing. Yonatan Sanz-Perl: Data curation; Investigation; Writing – original draft; Writing – review & editing. Enzo Tagliazucchi: Conceptualization; Investigation; Methodology; Supervision; Writing – original draft; Writing – review & editing. Morten Kringelbach: Supervision; Writing – review & editing. Rodrigo Cofré: Conceptualization; Formal analysis; Investigation; Methodology; Project administration; Supervision; Writing – original draft; Writing – review & editing. Gustavo Deco: Funding acquisition; Resources; Supervision; Writing – review & editing.

## FUNDING INFORMATION

Rubén Herzog, Agencia Nacional de Investigación y Desarrollo (https://dx.doi.org/10.13039/501100020884), Award ID: PFCHA/Doctorado Nacional/2018-21180428. Fernando Rosas, Ad Astra Chandaria Foundation (https://dx.doi.org/10.13039/501100022772). Gustavo Deco, HORIZON EUROPE European Research Council (https://dx.doi.org/10.13039/100019180), Award ID: 101071900.

## DATA AVAILABILITY STATEMENT

The C++ implementation of the FastDMF, its Python and MATLAB wrappers, and the corresponding datasets can be found at https://gitlab.com/concog/fastdmf.

## Supplementary Material



## TECHNICAL TERMS

Whole-brain model: Simulation of neural activity across the entire brain that defines local dynamics and interregional connectivity to reproduce empirical observations.
Dynamic Mean Field (DMF) model: A biophysical whole-brain model that leverages the mean-field approach to scale the population neural activity up to the whole-brain scale.
Feedback inhibition control (FIC): Local connection from the inhibitory to the excitatory population that regulates and stabilizes neural activity.
Parcellation: A specific division of the brain into distinct regions for analysis.
Resting-state fMRI recordings: Brain functional magnetic resonance imaging data collected during wakeful rest.
Functional connectivity: Statistical dependence between the temporal activity of a pair of brain regions recorded with neuroimaging recordings.
Functional connectivity dynamics (FCD): Temporal fluctuations in functional connectivity patterns between brain regions.
Structural connectivity: Anatomical network of inferred axonal connections linking brain regions.
Structural connectivity strength: The sum of all the structural connections of a given brain region.
