# Supplementary material for: Neural mass modeling for the masses: Democratizing access to whole-brain biophysical modeling with FastDMF
Source: Netw Neurosci. 2024 Dec 10;8(4):1590–612. doi: 10.1162/netn_a_00410 (PMC11674928; doi:10.1162/netn_a_00410)
Supplement: Supplementary file 1 [file netn-8-4-1590-s001.pdf]

Supporting information

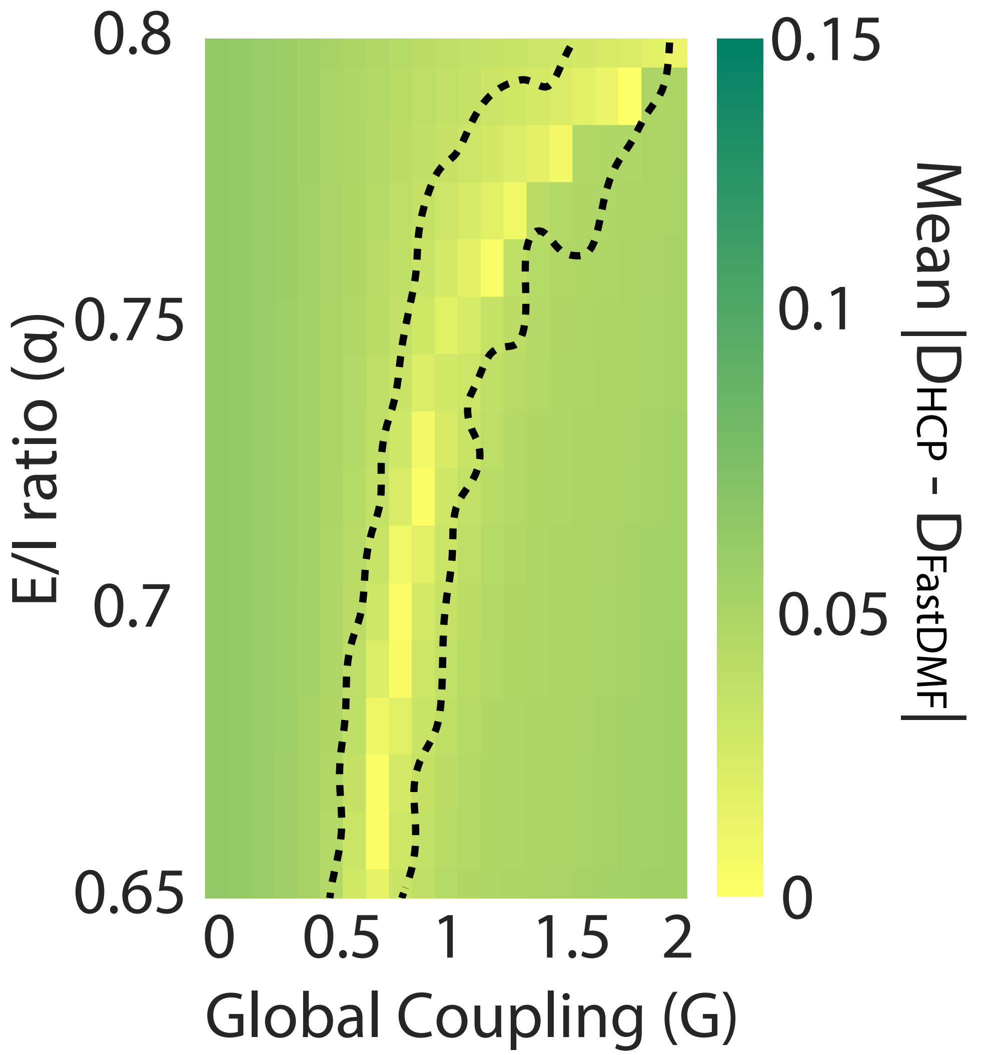

Supplementary Figure 1. Co-localization of best fit to FCD and to turbulence-like dynamics. Same as Figure 5A, but showing in dashed lines the best 10% fits to the FCD (from Figure 3E). Note that the data used for fitting the FCD was obtained from a different source than the HCP.
